# Endometrial Cancer Risk Prediction According to Indication of Diagnostic Hysteroscopy in Post-Menopausal Women

**DOI:** 10.3390/diagnostics10050257

**Published:** 2020-04-27

**Authors:** Carlo Saccardi, Amerigo Vitagliano, Matteo Marchetti, Alice Lo Turco, Sofia Tosatto, Michela Palumbo, Luciana Serena De Lorenzo, Salvatore Giovanni Vitale, Marco Scioscia, Marco Noventa

**Affiliations:** 1 Department of Women’s and Children’s Health, University of Padua, 35100 Padua, Italy; 2 Department of General Surgery and Medical Surgical Specialties, University of Catania, 95124 Catania, Italy; 3 Department of Obstetrics and Gynecology, Policlinico Abano Terme, 35031, Italy

**Keywords:** endometrial cancer, early diagnosis, endometrial thickness, diagnostic hysteroscopy, AUB, asymptomatic women, menopause

## Abstract

We conducted a prospective observational study investigating the clinical relevance of endometrial thickness (ET) and abnormal uterine bleeding (AUB) on endometrial cancer (EC) risk in a cohort of postmenopausal patients undergoing diagnostic hysteroscopy and endometrial biopsy. Patients were divided into two groups according to the indication of diagnostic hysteroscopy: ET_Group (asymptomatic patients with endometrial thickness ≥ 4 mm) and AUB_Group (patients with a history of abnormal uterine bleeding). We further divided the AUB_Group into two subgroups based on endometrial thickness (AUB_Subgroup1: ET < 4 mm; AUB_Subgroup2: ET ≥ 4 mm). The primary outcome was the risk of endometrial cancer and atypical hyperplasia according to the indications of diagnostic hysteroscopy (AUB, ET ≥ 4 mm or both). The secondary outcome was to determine the best cut-off value of endometrial thickness to predict endometrial cancer in asymptomatic postmenopausal women. The prevalence of endometrial cancer and atypical hyperplasia in AUB_Group and ET_Group was 21% and 6.7% respectively. As well as for EC alone, higher prevalence of both conditions was observed in AUB_Subgroup2 (29.3%) in comparison to AUB_Subgroup1 (10.6%; *p* < 0.001). In asymptomatic patients the cut-off of endometrial thickness that showed the best sensitivity and specificity to diagnose endometrial cancer (100% and 80% respectively) was 11 mm (AUC of 91.4%; Expβ: 1067; CI 95%). In conclusion, considering the high risk of neoplasia, diagnostic hysteroscopy with endometrial biopsy should be mandatory in cases of abnormal uterine bleeding in postmenopausal patients. Moreover, we want to emphasize the need for further evidence stating the clinical relevance of endometrial thickness value in asymptomatic patients and the impact of individual risk factors on endometrial cancer development.

## 1. Introduction

Endometrial cancer (EC) represents the most common and the second most lethal gynecological cancer in developed countries, with a lifetime risk of occurrence estimated at 2.5% [1,2].

EC mainly occurs in postmenopausal age, especially in the sixth and seventh decades of life. In about the 95% of cases the diagnosis is preceded by at least one episode of abnormal uterine bleeding (AUB), while the remaining patients are asymptomatic at the time of diagnosis [1,2,3].

The gold standard for diagnosis of EC is represented by endometrial biopsy (EB) with histopathological examination, preferably preceded by diagnostic hysteroscopy (HSC). However, due to its invasiveness, associated pain, risk of complications (0.3%, according to ACOG Committee opinion of March 2020, but considerable) and higher costs compared to ultrasound, HSC plus EB is not currently considered an effective strategy for EC screening in the general population [4]. However HSC is strictly recommended in the diagnostic management of patients with a high risk of cancer, such as women with AUB and “thickened endometrium” at pelvic ultrasound or with other risk factors (such as obesity, use of unopposed estrogen, atypical glandular cells on screening cervical cytology, family history of gynecologic malignancy, tamoxifen therapy) [5,6,7,8].

Differently from cervical cancer, whose impact on the global health has been completely changed since the introduction of the Papanicolau test, the search for an effective and reliable screening tool for EC is still in progress, even transvaginal ultrasonography alone seems appropriate [5,9,10,11]. Concerning asymptomatic postmenopausal women, endometrial thickness (ET) assessment through transvaginal ultrasound (TVUS) was celebrated by different authors as “the revolutionary tool” for EC screening due to minimal invasiveness, low costs and good repeatability [12]. Unfortunately, as stated by ACOG guidelines, TVUS should not be used as a screening tool for EC in asymptomatic women due to the low rate of cancer without bleeding. On the other hand, ACOG stated that TVUS is appropriate for an initial evaluation of postmenopausal bleeding, because an ET of 4 mm or lower seems to have a 99% negative predictive value for EC. We must consider that ET < 4 mm does not exclude all the disease and it remains unclear if an ET cut-off could also be associated with EC in patients without AUB [3,8,9,10,13,14].

As a consequence of such uncertainties and unanimity of opinions among clinicians, there follows an excessive and probably inappropriate use of diagnostic tools, with high health costs and a lack of improvement in the early diagnosis of EC [11].

In this regard, in order to help physicians in the decision-making process that leads to HSC plus EB, we conducted a prospective study investigating the clinical relevance of ET and AUB on the risk of EC in a cohort of postmenopausal patients undergoing HSC plus EB at our center. Moreover, we evaluated the correlation between the individual risk factors of BMI, hormone therapy (HT), hypertension and diabetes mellitus and the prevalence of EC.

## 2. Materials and Methods

### 2.1. Study Design

We conducted a prospective, observational study on a cohort of consecutive postmenopausal patients referred to Gynecological and Obstetrics Clinical Unit, Department of Women’s and Children’s Health, University of Padua/Hospital of Padua, for outpatient diagnostic hysteroscopy from June 2017 to June 2019. All patients signed a document approved by the hospital of Padua (protocol number 76359, Resolution of the executive director no. 1501) before the diagnostic procedure for the consent to the anonymous use of their clinical data for scientific purposes according to the European privacy law.

### 2.2. Inclusion and Exclusion Criteria

We considered eligible all patients with a history of natural menopause, defined as the spontaneous cessation of menses for at least twelve consecutive months after the age of forty years that could not be explained by medication or disease, in possession of a TVUS report with an ET measurement dated no more than three months before the diagnostic hysteroscopy. The patients were included in the study if they had an AUB history and/or an ET ≥ 4 mm at TVUS. TVUS reports were considered adequate only when the ET was measured, according to an ACOG statement, as the maximum anterior–posterior thickness of the endometrial echo on a long-axis transvaginal view of the uterus, with attached iconography relative to the measurement [8,9]. Patients were recruited on the day of outpatient admission before the diagnostic HSC.

Exclusion criteria were as follows: current or previous treatment with tamoxifen, aromatase inhibitors or Tibolone; unbalanced hormone therapy (HT); A TVUS report older than three months prior to HSC or without attached iconography; ET measurement not in accordance with ACOG guidelines; ongoing anticoagulant therapy; history of breast cancer; chemotherapy in the last twelve months. [15]

### 2.3. Data Collection and Definition of Groups

For each patient we recorded the indication to the exam, general features (age, BMI) and data about medical history (history of diabetes mellitus, hypertension previous balanced HT).

Diabetes mellitus was defined according to SID criteria (Italian society of Diabetology) [16]. Hypertension was defined according to recent ESC/ESH guidelines for the management of arterial hypertension, valid at the end of enlistment [17]. According to BMI values, patients were considered as normal-weight (BMI between 18–24.9 kg/m^2^), over-weight (BMI between 25–29.9 kg/m^2^) or obese (BMI ≥ 30 kg/m^2^).

For data analysis patients were split according to the indication for diagnostic HSC in ET_Group (asymptomatic patients with ET ≥ 4 mm) and AUB_Group (patients with history of AUB). Those included in AUB_Group were subsequently divided into two subgroups based on ET values (AUB_Subgroup1: ET < 4 mm; AUB_Subgroup2: ET ≥ 4 mm). All included patients underwent a diagnostic HSC with endometrial sampling in order to evaluate the uterine cavity and endometrial pattern (this is the standard of care in our unit in cases of menopausal patients with endometrium ≥ 4mm and/or AUB).

### 2.4. Diagnostic Hysteroscopy and Endometrial Sampling

Diagnostic HSC was performed in outpatient regimen with a vaginoscopic approach using a continuous-flow hysteroscope (Karl Storz—Karl Storz GmbH & Co., Tuttlingen, Germany) with saline solution (0.9% sodium chloride, pH 5.5) as a distension medium and 30° angle view optics (2.9 mm diameter).

The exploration of the uterine cavity consisted of a panoramic view of the cavity followed by a thorough evaluation of the endometrial pattern. In case of great discomfort or pain or internal cervical ostium stenosis, the procedure was suspended and subsequently performed in day surgery regimen under anesthesia.

The endometrial pattern was systematically evaluated according to a standard form and classified in normal hysteroscopy or suspected for a specific condition between benign lesion (polyp, myoma), low-risk hyperplasia, high-risk hyperplasia and carcinoma basing on a HYCA (hysteroscopic cancer) scoring system [18]. See Table 1 for details.

After hysteroscopy, an endometrial biopsy (EB) was obtained for each patient. In detail, in case of diffuse endometrial changes, a curettage of uterine cavity with Novak cannula was performed at the end of the hysteroscopic procedure, while the focal lesions were removed using a resectoscope at a later time. All endometrial specimens were sent to the pathologist and classified in three histopathological categories: no disease (atrophic endometrium or benign lesion); AH (atypical hyperplasia); EC (endometrial cancer).

The findings on microscopic analysis of resectoscopic biopsy specimens and the Novak cannula samples at hysteroscopy served as the reference standard for statistical analysis.

### 2.5. Outcomes of the Study

The primary outcome of our study was to assess the risk of endometrial cancer and atypical hyperplasia in relation to the indication for diagnostic HSC (AUB, ET ≥ 4 mm or both).

The secondary outcome was to determine the best cut-off value of ET for the prediction of EC in asymptomatic postmenopausal women (ET_Group—asymptomatic patients with ET ≥ 4 mm).

The third outcome was to evaluate the impact of overweight (BMI ≥ 25 kg/m^2^), hypertension, diabetes and EPT (estrogen plus progestin therapy) on the risk of EC according to the indication for HSC.

### 2.6. Statistical Analysis

Data were analyzed by SPSS 22.0 software (SPSS Inc, Chigaco, IL). A statistical power analysis was performed for sample size estimation. Considering an estimated prevalence of EC or AH in postmenopausal asymptomatic patients with ET ≥ 4 mm of about 3% and an estimated prevalence of about 15% in postmenopausal patients with AUB, applying an alpha = 0.05 and a power = 0.80 with a 1:1 ratio, we calculated that a minimum sample size of 86 per group could be adequate for the main endpoint of the study; considering a possible drop out of about 10%, we decided to set a minimum sample size of 95 patients per group [19,20].

Continuous variables were calculated as mean plus or minus the standard deviation, whereas qualitative variables were presented as absolute frequencies and percentages. Comparisons between categorical variables were tested by using contingency tables and chi-square test or Fisher’s test when necessary. Comparisons between normally distributed continuous variables were performed by using Student’s t-test. The Mantel-Haenszel method was used to estimate relative risk (RR). Receiver operating characteristic (ROC) curves were elaborated to assess the performance of ET to detect EC among asymptomatic women. Multiple logistic regression was used to identify the effect of BMI (normal weight versus overweight/obese), hypertension, diabetes mellitus and endometrial thickening on EC diagnosis. Statistical significance was determined using two-tailed *p*-values, and the significance level was set at *p* < 0.05.

## 3. Results

### 3.1. Patients’ Characteristics

Between June 2017 and June 2019, 903 postmenopausal patients referred to the Obstetrics and Gynecological Clinic of Padua for diagnostic HSC, of which 518 were assessed for eligibility. Among 518 patients, 84 were excluded for inadequate or outdated TVUS documentation, while 435 were included in the study.

**Figure 1:**
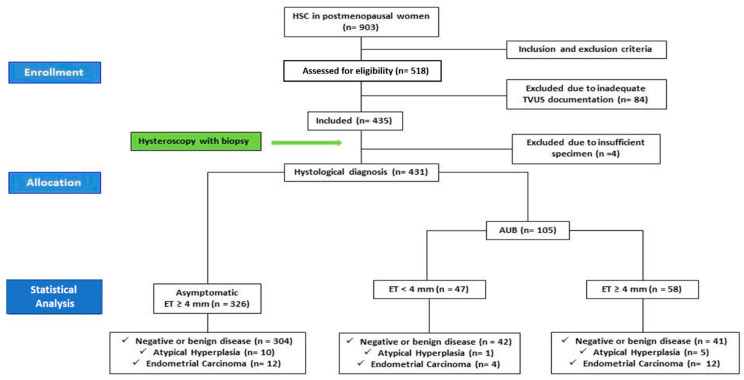
Flow chart of the study.

Among these 435 women, 329 were asymptomatic and were referred to diagnostic HSC due to ET ≥ 4 mm (ET_Group). One hundred and six patients received indication to HSC due to AUB (AUB_Group), of which 48 were with ET < 4 mm (AUB_Subgroup1) and 58 were with ET ≥ 4 mm (AUB_Subgroup2). After histological examination, four patients (three patients of ET_Group and one patient of AUB_Group) were excluded due to insufficient specimens. Therefore, 431 patients were considered for the statistical analysis. See Figure 1 for details.

Mean age was 63.8 ± 9.3 years and mean BMI was 26.07 ± 5.31 kg/m^2^. Overweight (BMI ≥ 25 kg/m^2^) and hypertension were identified respectively in 22.5% and 32% of the study population. See Table 2 for details.

### 3.2. Risk of Endometrial Cancer and Atypical Hyperplasia

From the analysis of histological specimens, we found a prevalence of EC of 3.7% (12 patients) in ET_Group, versus a prevalence of 15.2% (16 patients) in AUB_Group (*p* < 0.001). Histological examination all resulted in endometrioid endometrial adenocarcinoma. The RR for diagnosing EC in case of AUB was estimated to be 4.1 (CI 95% 2.02 to 8.46).

In AUB_Group, among the 16 women with a diagnosis of EC, 4 patients were observed in AUB_Subgroup1 and 12 in AUB Subgroup2, with a significantly higher prevalence of EC in women with ET ≥ 4 mm and AUB rather than AUB alone (20.7% vs 8.5%; *p* < 0.001).

Next, we analyzed contextually the diagnosis of both EC and AH into the two groups. We observed respectively a prevalence of 21% (22 patients; *p* > 0.001) in AUB_Group and 6.7% (22 patients; *p* > 0.001) in ET_Group.

The RR for diagnosing EC and AH in cases of AUB was estimated to be 3.1 (CI 95% 1.79 to 5.37). Moreover, as well as for EC alone, higher prevalence of both conditions was observed in AUB_Subgroup2 (29.3%; 17 patients) in comparison to AUB_Subgroup1 (10.6%; 5 patients; *p* < 0.001). See Table 3 for details.

### 3.3. Diagnostic Test Accuracy

The estimation of the risk of EC in relation to ET alone (ET_Group), obtained through a ROC curve analysis, showed an AUC of 91.4% (Expβ: 1.067; CI 95%). The cut-off with the best sensitivity and specificity for EC diagnosis (respectively 100% and 80%) resulted 11 mm, with a progressive increase of EC risk to the value of 30 mm (*p* = 0.001). Overcoming the value of 30 mm, the risk of EC did not show any further increase.

AUB_Group sensitivity and specificity of transvaginal ultrasound for EC screening (with 4 mm cut-off to define an abnormal endometrial thickening) resulted respectively 75% (CI 95% 47.62% to 92.73%) and 48.3% (CI 95% 37.59% to 59.16%).

### 3.4. Impact of Other Factors on the Risk of EC

Concerning the connection between EC and the risk factors ET, BMI, hypertension, diabetes and EPT, in the multiple logistic regression model we found a correlation only for ET (Expß 1.067, *p* = 0.015) and BMI ≥ 25 kg/m^2^ (Expβ 6.826, *p* = 0.019), while for the other variables we did not observe significant results.

## 4. Discussion

### 4.1. General Considerations

In the last twenty years, the increased life expectancy of women has led to a significant rise in the social impact of EC, with about 200,000 new diagnoses and 50,000 deaths worldwide each year [21]. According to ACOG guidelines, transvaginal ultrasonography is a reasonable alternative to endometrial sampling as a first approach in evaluating a postmenopausal woman with an initial episode of bleeding if prior probability of cancer and hyperplasia is low. In these women, an endometrial thickness of 4 mm or less has a greater than 99% negative predictive value for detecting EC [8]. These findings were confirmed by Long et al. in a recent meta-analysis that proposed an endometrial thickness cut-off of ≥ 5 mm as good enough to rule out the presence of EC in patients with AUB [22]. However, different authors raised concerns about an approach based only on ET-specific cut-offs to exclude the presence of EC and our results support these findings [23,24,25].

### 4.2. Main Findings

In line with our previous experience, although patients with ET ≥ 4 mm showed a significantly higher prevalence of EC in comparison to women with ET < 4 mm, the overall accuracy of ET in the detection of cancer appeared too poor, precisely with 75% sensitivity and 48.3% specificity. Moreover, we found an alarmingly high prevalence of EC in symptomatic patients with ET < 4 mm (8.5%), suggesting that the value of ET in patients with AUB probably needs to be further explored, or eventually needs to be combined with other risk factors in a new diagnostic score for EC. In this regard, Long et al., at the end of his meta-analysis, considered some clinical EC risk scoring systems, but all of them resulted in lower sensibility and specificity than TVUS alone using ET cut-off ≥ 5 mm [22].

In this regard we need to underline that there is lack of consensus about the best ET “warning cut-off” to use (3, 4 or 5 mm) for the selection of patients with AUB that require an EB; in addition, none of the suggested cut-off showed a sensitivity of 100% [10,11,22,26]. In particular, Long et al. showed that ET cut-offs of ≥ 3 mm, ≥ 4 mm, ≥ 5 mm all seemed sensitive for EC among women with AUB history, and therefore he supported the use of an ET cut-off of 5 mm due to the higher specificity [22]. On the other hand, some authors believed that 4–5% false negative rate in cancer detection was unacceptable in symptomatic women. [25]

It is also universally known that TVUS is an operator-dependent technique, with a significant inter- and intra-operator variability and non-negligible bias especially in small measurements, in which even a single millimeter, more or less, may be discriminative for the subsequent clinical choice [27].

For all these reasons we believe that, waiting for further evidence about ET safety, its clinical significance should be considered with caution and probably not absolutely discriminative for biopsy in patients with AUB, particularly for high-risk patients or patients with recurrent or persistent AUB [28]. In fact, ACOG recommended that ultrasonography should be used only for patients with initial AUB whose prior probability of cancer and hyperplasia is low enough that no additional testing would be required after a normal ultrasonography. Endometrial sampling should be the first-line test for women with postmenopausal bleeding at higher risk (based on clinical risk factors or clinical presentation). Moreover, women with persistent or recurrent bleeding should trigger additional evaluation, such as endometrial sampling and hysteroscopy [8]. A suggestion for further studies could be the use of ET as a tool to define the urgency of EB in women with postmenopausal AUB [23,29].

Different international societies have stated that no screening for EC results are recommended in asymptomatic postmenopausal patients, and so the use of ET measurement should be avoided in the evaluation of EC risk [8,9,10,11].

However, although there is general consensus in discouraging the measurement of ET in this group of patients, physicians commonly deal with a clinical dilemma when they have to manage a patient with an “incidentally discovered thickened endometrium” during a TVUS performed for other reasons.

Indeed, to date, the clinical significance of ET in this group of patients has not been completely elucidated despite the important step forward in non-invasive gynecologic diagnostics and in mini-invasive gynecologic surgery, resulting in an overflow of postmenopausal patients to diagnostic HSC services and a rise in health care costs [11,30,31]. Due to a lack of unanimous international consensus, our daily clinical practice is to offer a diagnostic HSC both in cases of AUB and in cases of an incidental discovery of ET ≥ 4 mm. In fact, we found that the incidental finding of ET ≥ 4 mm represented the main indication for diagnostic HSC in our center (75.6% of the study population), with only a small prevalence of EC (3.68%) and AH (3.07%) and a high percentage of negative biopsy (93.25%).

According to SOGC and ACOG, in the light of our experience, we believe that a threshold of 4 mm, commonly used in cases of patients with AUB, should not be extrapolated to asymptomatic patients due to poor positive predictive value [8]. Indeed, the incidence of ET ≥ 4 mm in asymptomatic postmenopausal women have been reported to be up to 17%, extremely high in comparison to the incidence of endometrial cancer (lower than 2/1000) [4,8,11]. In this regard, according to Smith-Bindman et al., we interestingly found a significant risk of cancer only for values of ET considerably higher than 4 mm, starting from 11 mm [14,32]. Intriguingly, the cut-off value 11 mm showed a 100% sensitivity and 80% specificity for EC detection, with a progressive increase of cancer risk for ascending values up to 30 mm. The cause of the observed stop in the growing trend of risk for values higher than 30 mm was probably due to the unlikelihood of a “such thick endometrium” to be asymptomatic in the case of cancer, as opposed to some benign lesions as large polyps, which may sometimes not correlate with AUB.

Moreover, an interesting finding emerged from our analysis of the correlation between some individual risk factors and EC prevalence [33,34]. We found a strong correlation between EC and elevated BMI, while no increase of risk emerged in patients with hypertension, diabetes or a history of EPT, different from the findings of Maatela et al. and other authors. [35,36,37].

In detail, we found that overweight (including obese) patients (with BMI ≥ 25 kg/m^2^) were much more likely to be affected by EC in comparison to normal-weight patients, suggesting that BMI has not only a weight on the surgical time and overall survival of patients with EC, but should also be considered in the decision-making process of both symptomatic and asymptomatic postmenopausal women to nominate them for HSC plus EB [38,39,40,41,42].

### 4.3. Strengths and Limitations

The strength of our study is represented by the prospective enrolment of patients and the accurate study design with strict inclusion and exclusion criteria. At the same time, although we enrolled a relevant number of patients, most of them were asymptomatic which therefore led to a substantial difference in sample size among ET_group and AUB_group, with a limited number of cancer cases.

Diagnostic Hysteroscopies and biopsies were all performed by the same operator (CS) with several years of experience in hysteroscopy. On the other hand, endometrial thickness assessment was not performed by the same operator and that could lead to differences in its evaluation, since TVUS is an operator-dependent technique, with a significant inter- and intra-operator variability and non-negligible bias, especially in small measurements.

## 5. Conclusions

In our opinion, the present study provides useful information for physicians, adding new and interesting insights into the risk assessment for EC in relation to the indication of the execution of diagnostic HSC plus biopsy. In particular, considering the increased risk of cancer in asymptomatic postmenopausal patients with ET ≥ 11, we want to emphasize the need for further evidence stating the clinical relevance of ET value in this group of patients and the impact of individual risk factors on the risk of EC. With Breijer et al., we sincerely believe that the choice of the best diagnostic approach for EC cannot disregard a careful assessment of a patient’s characteristics that should be evaluated case by case, in the view of a “personalized diagnostic workup” [11,24,30,34,43].

Finally, our experience puts a question mark about the safety of ET in excluding cancer in patients with AUB, raising the reasonable assumption that diagnostic HSC with EB might always be performed in this group of patients.

## Figures and Tables

**Table 1:** Endometrial evaluation during hysteroscopy.

Variable	Pattern Characteristics
Generality	Atrofic Thickened Uneven
Surface	Regular Polypoid (Local v.s. Diffuse) Papillary projections (Local v.s. Diffuse) Irregular surface (Local v.s. Diffuse)
Necrosis	No evidence Surface necrosis Candy floss pattern Hyperintense white spots
Vessel pattern	Regular Irregular branching Irregular distribution
Gland pattern	Regular Dilated glands and glands with irregular openings
Suspected for	Regular Low-risk hyperplasia High-risk hyperplasia Adenocarcinoma

**Table 2:** General features of patients included in the study.

Variable	Frequency
Age (y)	63.8 (±9.3)
BMI (kg/m^2^)	26.07 (±5.31)
Overweight (*n*)	114 (26.5%)
Obesity (*n*)	97 (22.5%)
Hypertension (*n*)	138 (32%)
Diabetes Mellitus (*n*)	63 (15%)
Hormone Replacement Therapy (*n*)	77 (17.9%)

**Table 3:** Prevalence of endometrial cancer and atypical hyperplasia according to Groups.

**Groups**	**EC**	**Negative**	***p* Value**
ET_Group (*n* = 326) (ET ≥ 4 mm asymptomatic)	12 (3.7%)	314 (96.3%)	<0.001
AUB Group (*n* = 105)	16 (15.2%)	89 (84.8%)	
AUB Subgroup 1 (*n* = 47) (ET < 4 mm)	4 (8.5%)	43 (91.5%)	<0.001
AUB Subgroup 2 (*n* = 58) (ET ≥ 4 mm)	12 (20.7%)	46 (79.3%)	
	**EC and Atypical Hyperplasia**	**Negative**	***p* Value**
ET_Group (*n* = 326) (ET ≥ 4 mm asymptomatic)	22 (6.7%)	304 (93.3%)	<0.001
AUB Group (*n* = 105)	22 (21.0%)	83 (79.0%)	
AUB Subgroup 1 (*n* = 47) (ET < 4 mm)	5 (10.6%)	42 (89.4%)	<0.001
AUB Subgroup 2 (*n* = 58) (ET ≥ 4 mm)	17 (29.3%)	41 (70.7%)	

**Legend**: ET (endometrial thickness); EC (endometrial cancer); AUB (abnormal uterine bleeding).
